# Hip Pain Associated with Acetabular Dysplasia in Patients with Suspected Axial Spondyloarthritis: DESIR Cohort Data

**DOI:** 10.1186/s12891-022-05575-4

**Published:** 2022-07-05

**Authors:** Dewi Guellec, Guillaume Prado, Corinne Miceli-Richard, Guillermo Carvajal-Alegria, Alain Saraux

**Affiliations:** 1 Service de rhumatologie, CHU Brest, INSERM CIC 1412, Brest, France; 2 Service de rhumatologie, Département de médecine du sport, CHU Brest, Brest, France; 3 Service de rhumatologie, Hôpital Cochin, Université de Paris, Paris, France; 4 Service de rhumatologie, CHU Brest, Hôpital La Cavale Blanche, INSERM 1227, Université de Bretagne Occidentale, LabEx IGO, Bd. Tanguy Prigent, 29200 Brest, France

**Keywords:** Axial Spondyloarthritis, Acetabular dysplasia, Hip

## Abstract

**Objectives:**

To determine whether acetabular dysplasia is associated with hip pain at physical examination among adults with recent-onset inflammatory back pain (IBP) suggesting axial spondyloarthritis (axSpA).

**Methods:**

This cross-sectional ancillary study was conducted on the prospective DESIR cohort, which enrolled patients aged 18–50 years who had recent-onset IBP. Two readers used antero-posterior pelvic radiographs to assess the Tönnis angle, acetabular angle (AA), lateral centre-edge angle (LCEA), and femoral head extrusion index (FHEI). Abnormality of one or more of these four variables defined acetabular dysplasia. Hip pain upon physical examination was assessed based on Ritchie’s articular index.

**Results:**

The overall prevalence of acetabular dysplasia was 22% (139/636). The proportion of females was higher in the group with acetabular dysplasia. Hip pain was found in 21% (29/139) of patients with versus 12% (59/497) without acetabular dysplasia (OR, 1.96; 95% CI, 1.20 to 3.20); the association was significant in males (OR, 3.14; 95% CI, 1.44 to 6.86) but not females (OR, 1.39; 95% CI, 0.74 to 2.62). Results were similar when acetabular dysplasia was defined on the basis of LCEA alone (OR, 2.15; 95% CI, 1.18 to 2.62).

**Conclusion:**

Among patients with recent-onset IBP suggesting axSpA, acetabular dysplasia was significantly associated with hip pain in males. Hip pain related to acetabular dysplasia might result in overdiagnosis of hip involvement by axSpA.

## Key messages

**What is already known about this subject?**

- Undiagnosed mild acetabular dysplasia is common in adults and may cause hip pain.
- Axial spondyloarthritis is challenging to diagnose and can involve the hips.

**What does this study add?**

- In patients with recent-onset inflammatory back pain suggesting axial spondyloarthritis, the common disorder of acetabular dysplasia was significantly associated with hip pain upon physical examination in males but not in females.

**How might this impact on clinical practice or future developments?**

- Acetabular dysplasia should be sought routinely in patients with suspected axial spondyloarthropathy and, if present, should be considered among the possible causes of hip pain.

## Introduction

Axial spondyloarthritis (axSpA) is a chronic inflammatory rheumatic disease that predominantly affects the axial skeleton, causing inflammatory back pain (IBP) [1]. The diagnosis and treatment may raise challenges, as many patients have associated conditions that produce overlapping symptoms and signs. In patients with inflammatory joint disease, common conditions responsible for joint symptoms and/or functional impairment may mistakenly suggest treatment failure by increasing disease activity scores or the global burden of symptoms [2, 3]. Better identification of these associated conditions should improve the management of patients with suspected or confirmed axSpA by avoiding overtreatment and/or resulting in specific treatments [4].

Inflammatory hip involvement is common in axSpA. In a retrospective single-centre observational study, radiological evidence of inflammatory hip disease was found in 18% of patients with axSpA, often within the first few years after the diagnosis and was mostly bilateral [5]. In addition, joint replacement surgery was required in a third of patients with hip involvement, suggesting an association with greater axSpA severity [5]. Hip involvement must therefore be identified early, to ensure that treatment is optimal. In patients with hip pain but no radiological evidence of hip inflammation and/or no joint effusion, the possibility that another disease is causing the hip symptoms should be assessed to avoid overtreatment of axSpA.

Conditions that can cause unilateral or bilateral hip pain in adults include tendinitis, osteoarthritis, impingement syndrome, and acetabular dysplasia. Acetabular dysplasia is common in adults (overall prevalence ranging from 5 to 25% according to population and definition) and can cause pain even before the common complication of premature osteoarthritis [6–8]. Pain due to mild acetabular dysplasia may not be sufficiently severe to lead patients to seek medical advice. In patients undergoing an overall articular assessment, for instance for suspected axSpA, the presence of acetabular dysplasia may generate confusion about the source of the symptoms.

Here, our objective was to determine whether radiological acetabular dysplasia was associated with hip pain upon physical examination in adults with recent-onset IBP suggesting axSpA and might, therefore, mistakenly suggest clinical hip disease related to axSpA.

## Methods

The prospective DESIR (*DEvenir des Spondylarthropathies Indifférenciées Récentes*, outcomes of recent-onset undifferentiated spondyloarthritis) cohort included 708 patients aged 18–50 years who had IBP for at least 3 months with onset within the past 3 years and symptoms suggesting axSpA according to the local investigator [9]. Patients were included in 25 centers throughout France between December 2007 and April 2010 and were monitored prospectively for at least 5 years. The DESIR study was approved by the appropriate ethics committee and was conducted according to good clinical practice guidelines.

Clinical data collected at baseline and relevant to the present ancillary study included age, sex, body mass index (BMI), smoking history, and ethnicity. Presence of hip pain at physical examination was prospectively assessed by determining the Ritchie Articular Index (RAI) for both hips, with 0 indicating no tenderness; 1, pain reported by the patient; 2, reported pain and wincing; and 3, reported pain, wincing, and effort to withdraw [10]. Significant hip pain upon physical examination was defined as RAI ≥ 1. We recorded the criteria used to classify axSpA according to Amor [11], the European Spondyloarthropathy Study Group (ESSG) classification [12], and the Assessment in SpondyloArthritis international Society (ASAS) [13, 14]. For the assessment of axSpA disease activity and functional impairment, we collected the C-reactive protein (CRP) level, Ankylosing Spondylitis Disease Activity Score (ASDAS), Bath Ankylosing Spondylitis Disease Activity Index (BASDAI), and Bath Ankylosing Spondylitis Functional Index (BASFI). All patients underwent antero-posterior pelvic radiographs at baseline, as well as radiographs and magnetic resonance imaging (MRI) of the sacro-iliac joints. Patients were routinely evaluated for sacro-iliitis according to ASAS criteria and to the modified New York classification (mNY) [15]. As patients did not undergo profile radiographs, assessment of the anterior coverage of the femoral heads was not possible.

**Fig. 1:**
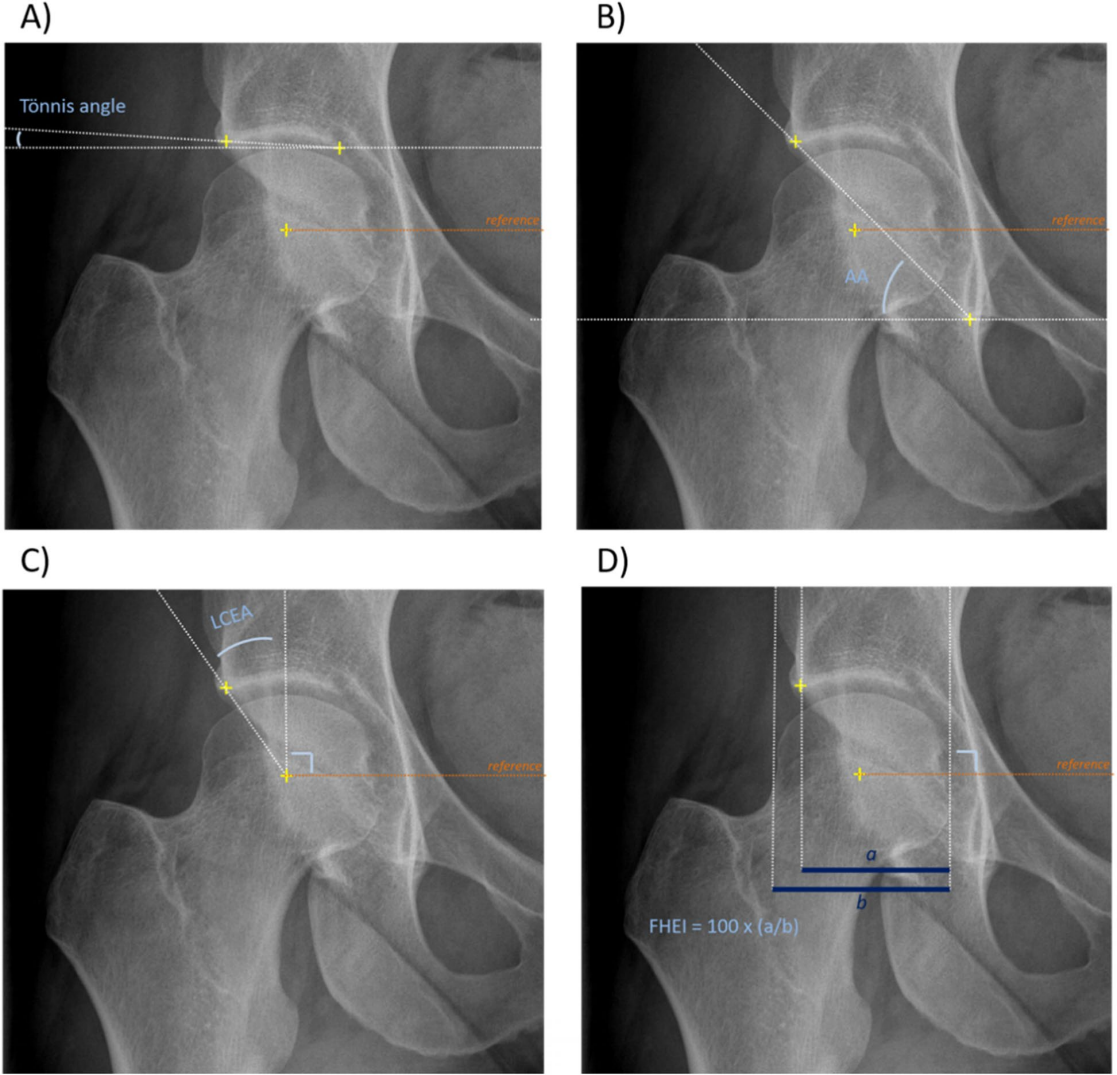
Parameters used to define acetabular dysplasia. **A** Tönnis angle, **(B)** acetabular angle (AA), **(C)** lateral centre edge angle (LCEA), **(D)** femoral head extrusion index (FHEI). The orange line passes through the centre of the femoral heads

Patients whose antero-posterior pelvic radiographs allowed a valid assessment of both hips and who had no history of hip surgery were considered for the present study and assessed for acetabular dysplasia, using widely accepted morphological parameters. All radiographs were evaluated twice, by two experienced rheumatologists (DG and GP), who were blinded to all other study data. As a preliminary step, reliability and repeatability of the assessment were evaluated using an independent dataset of 50 antero-posterior radiographs including 25 anonymised radiographs from patients with acetabular dysplasia and 25 anonymised radiographs from consecutive patients admitted for sciatica. To measure the Tönnis angle, acetabular angle (AA), lateral centre-edge angle (LCEA), and femoral head extrusion index (FHEI) (Fig. 1) [16] on DICOM-format images, the readers used a specific macro implemented via ImageJ software [17]. Users were asked to position the medial and lateral edges of the acetabular sourcil as well as the inferior margin of the pelvic teardrop, at both hips. They also positioned at least 7 points on the outline of the femoral head in order to accurately determine the coordinates of its center. The line passing through the inferior margin of both pelvic teardrops was chosen, as an alternative to the horizontal line, to serve as the reference for measurements.

Values of the parameters were calculated automatically from these data. For each parameter measured in DESIR-cohort patients, the mean of the two values obtained by each independent reader was taken as the reference. Cut-offs used to define acetabular dysplasia were Tönnis angle > 12°, AA> 45°, LCEA< 20°, and FHEI< 70%, in agreement with many previous studies [16]. At the hip level and patient level, having at least one abnormal parameter defined acetabular dysplasia. Radiographs were also evaluated by both readers for the presence of hip osteoarthritis stage 2 or higher in the Kellgren-Lawrence classification [18], including final adjudication of discordant cases.

### Statistical analysis

In the dataset of 50 radiographs, inter-observer and intra-observer reproducibility of radiographic parameter measurement was evaluated by computing the intraclass correlation coefficients (ICC), which were interpreted as follows: < 0.50, poor; 0.50 to < 0.75, moderate; 0.75 to < 0.90, good; and ≥ 0.90, excellent [19].

The variables from DESIR-cohort patients were described as mean ± SD if continuous and n (%) if categorical. We reported the prevalence of acetabular dysplasia and of each abnormal acetabular-dysplasia parameter at the patient level and hip level, overall and separately for females and males. Prevalence of hip pain upon physical examination and radiological hip osteoarthritis were also reported. The groups with acetabular dysplasia (i.e., with at least one abnormal acetabular-dysplasia parameter) and without acetabular dysplasia were compared regarding general characteristics and features relevant to the diagnosis of axSpA. Association between acetabular dysplasia and hip pain upon physical examination was then assessed, at the patient level and hip level, considering assessment at the patient level as the main objective of the study. Given that LCEA is the most consensual indicator of acetabular dysplasia, we then conducted similar analyses with acetabular dysplasia defined as LCEA< 20°. Finally, we performed sub-groups analyses in groups defined by sex and age (18–24 years, 25–34 years, 35–44 years, and ≥ 45 years). Differences were assessed using the Mann-Whitney test for continuous variables and the χ^2^ test or Fisher’s exact test, as appropriate, for categorical variables. We performed logistic regression to ensure that significant associations found at individual level persisted after adjusting on main potential confounders for which data were available (age, sex and BMI). Values of *p* smaller than 0.05 were taken to indicate statistically significant differences. The statistical analyses were performed using SPSS software version 23.0 (IBM, Armonk, NY).

## Results

### Patient characteristics

Among the 708 patients in the DESIR cohort, 636 had baseline antero-posterior pelvic radiographs that allowed valid measurement of acetabular-dysplasia parameters, 341 (53.6%) females and 296 (46.4%) males. Mean age at inclusion was 33.7 ± 8.5 years and mean BMI was 23.9 ± 4.7 kg/m^2^.

The ICC values indicated excellent intra-observer and inter-observer reproducibility of radiographic measurements at both hips (≥ 0.93), except for AA at the left hip, for which reproducibility was good (inter-observer ICC = 0.78; intra-observer ICC = 0.86). Of the 636 patients, 139 (21.9%) had acetabular dysplasia at one or both hips. The prevalence was 26.4% (90/341) in females and 16.6% (49/295) in males. Table 1 reports additional details related to parameters assessing acetabular dysplasia at both sides. The only significant difference between the two groups, regarding general and axSpA characteristics, was a higher proportion of females in the group with acetabular dysplasia (Table 2).

**Table 1:** Prevalence of acetabular dysplasia among 636 patients of the DESIR cohort. The data are numbers (percentages).

	Right hip	Left hip	Right and/or left hip
**Tönnis angle > 12°**			
Females	29/341 (8.5%)	17/341 (5.0%)	35/341 (10.3%)
Males	21/295 (7.1%)	16/295 (5.4%)	26/295 (8.8%)
Total	50/636 (7.9%)	33/636 (5.2%)	61/636 (9.6%)
**Acetabular angle (AA) > 45°**			
Females	50/341 (14.7%)	30/341 (8.8%)	58/341 (17.0%)
Males	14/295 (4.7%)	5/295 (1.7%)	15/295 (5.1%)
Total	64/636 (10.1%)	35/636 (5.5%)	73/636 (11.5%)
**Lateral centre-edge angle (LCEA) < 20°**			
Females	38/341 (11.1%)	28/341 (8.2%)	48/341 (14.1%)
Males	20/295 (6.8%)	12/295 (4.1%)	24/295 (8.1%)
Total	58/636 (9.1%)	40/636 (6.3%)	72/636 (11.3%)
**Femoral head extrusion index (FHEI) < 70%**			
Females	34/341 (10.0%)	17/341 (5.0%)	43/341 (12.6%)
Males	18/295 (6.1%)	16/295 (5.4%)	28/295 (9.5%)
Total	52/636 (8.2%)	33/636 (5.2%)	71/636 (11.2%)
**Any parameter consistent with acetabular dysplasia**			
Females	77/341 (22.6%)	45/341 (13.2%)	90/341 (26.4%)
Males	40/295 (13.6%)	27/295 (9.2%)	49/295 (16.6%)
Total	117/636 (18.4%)	72/636 (11.3%)	139/636 (21.9%)

**Table 2:** Characteristics of the DESIR-cohort participants with and without acetabular dysplasia

	Acetabular dysplasia (***n =*** 139)	No acetabular dysplasia (***n =*** 497)	***p*** value^**a**^
**General characteristics**			
Age (years), mean ± SD	32.8 ± 8.9	34.0 ± 8.4	0.14
Female, n (%)	90 (64.7)	251 (50.5)	0.003
Body mass index, mean ± SD	23.6 ± 3.9	24.0 ± 4.2	0.60
Active smokers, n (%)	53 (38.1)	182 (36.6)	0.88
**Classification criteria for SpA, n (%)**			
ASAS criteria	93 (67.4)^b^	313 (63.7)^c^	0.43
ESSG criteria	116 (83.5)	387 (77.9)	0.15
Amor criteria	114 (82.0)	384 (78.2) ^c^	0.18
**Disease activity, mean ± SD**			
ASDAS-CRP	2.6 ± 0.9	2.6 ± 1.0	0.92
BASDAI	45.1 ± 19.2	44.1 ± 20.7	0.66
C-reactive protein (mg/L)	6.8 ± 10.6	8.2 ± 14.7	0.70
**Functional impairment** BASFI, mean ± SD	32.7 ± 23.3	29.8 ± 22.8	0.18
**Radiological involvement**			
Radiographic sacroiliitis (mNY criteria)	22 (15.8)	83 (16.7)	0.81
Sacroiliitis by MRI	47/ (34.1)^b^	166 (34.2)^d^	0.98

^a^Mann-Whitney test or χ^2^ test, as appropriate

^b^Data missing for 1 patient

^c^Data missing for 6 patients

^d^Data missing for 11 patients

*SpA* Spondyloarthritis, *ASAS* Assessment in SpondyloArthritis international Society, *ASDAS* Ankylosing Spondylitis Disease Activity, *BASDAI* Bath Ankylosing Spondylitis Disease Activity Index, Bath Ankylosing Spondylitis Functional Index, *ESSG* European Spondyloarthropathy Study Group, *mNY* Modified New York, *MRI* Magnetic resonance imaging

Overall, the RAI indicated unilateral or bilateral hip pain at the baseline physical examination in 88 (13.8%) patients. Right hip pain was found in 64 patients and left hip pain in 52 patients. The analysis by sex showed hip pain in 53 (15.5%) females and 35 (11.9%) males.

Hip osteoarthritis was present in 8 (1.3%) patients at baseline; all 8 had osteoarthritis of the right hip and 3 also had osteoarthritis of the left hip. Of these 8 patients, 3 (37.5%) had at least one abnormal acetabular-dysplasia parameter. Of the 8 patients with radiological hip osteoarthritis, only 1 had hip pain.

### Association of acetabular dysplasia with hip pain

In the groups with vs. without acetabular dysplasia of the right and/or left hip, the prevalence of hip pain at the baseline physical examination was 20.9% (29/139) and 11.9% (59/497), respectively (OR, 1.96; 95% CI, 1.20 to 3.20; *p =* 0.007). Logistic regression analysis showed that this association persisted after adjusting for age, sex and BMI (aOR, 1.89; 95% CI, 1.15 to 3.10; *p =* 0.01). When we separately assessed the patients with RAI values of 1, 2, and 3, we found that the prevalence of acetabular dysplasia was 26.3% (15/57), 44.0% (11/25), and 50.0% (3/6), respectively. The analysis by sex showed that acetabular dysplasia was significantly associated with hip pain in males (OR, 3.14; 95% CI, 1.44 to 6.86; *p =* 0.003) but not in females (OR, 1.39; 95% CI, 0.74 to 2.62. *p =* 0.31) (Table 3). The association found in males persisted after adjusting for age and BMI (aOR, 3.19; 95% CI, 1.45 to 7.04; *p =* 0.004). At the hip level, the presence of acetabular dysplasia was significantly associated with pain. The findings were similar when LCEA< 20° was required to define acetabular dysplasia in the whole population, for both unadjusted (OR, 2.15; 95% CI, 1.18 to 2.62. *p =* 0.01) and adjusted analysis (aOR, 2.13; 95% CI, 1.17 to 3.88; *p =* 0.00X). In contrast, the association between acetabular dysplasia and hip pain was similar in all age groups.

**Table 3:** Association of acetabular dysplasia with hip pain at physical examination in 636 patients of the DESIR cohort

	Right hip acetabular dysplasia (any parameter^**a**^)			Left hip acetabular dysplasia (any parameter ^**a**^)			Right and/or left hip acetabular dysplasia (any parameter ^**a**^)		
	Yes	No	***p*** value	Yes	No	***p*** value	Yes	No	***p*** value
**Right hip pain at physical examination**									
Females	11/77 (14.3%)	25/264 (9.5%)	0.23	7/45 (15.6%)	29/296 (9.8%)	0.24	12/90 (13.3%)	24/251 (9.6%)	0.32
Males	6/40 (15.0%)	22/255 (8.6%)	0.20	6/27 (22.2%)	22/268 (8.2%)	0.03	7/49 (14.3%)	21/246 (8.5%)	0.21
Total	17/117 (14.5%)	47/519 (9.1%)	0.08	13/72 (18.1%)	51/564 (9.0%)	0.02	19/139 (13.7%)	45/497 (9.1%)	0.11
**Left hip pain at physical examination**									
Females	9/77 (11.7%)	23/264 (8.7%)	0.43	4/45 (8.9%)	28/296 (9.5%)	1.0	9/90 (10.0%)	23/251 (9.2%)	0.82
Males	9/40 (22.5%)	11/255 (4.3%)	< 0.001	8/27 (29.6%)	12/268 (4.5%)	< 0.001	10/49 (20.4%)	10/246 (4.1%)	< 0.001
Total	18/117 (15.4%)	34/519 (6.6%)	0.002	12/72 (16.7%)	40/564 (7.1%)	0.005	19/139 (13.7%)	33/497 (6.6%)	0.008
**Hip pain at physical examination**									
Females	16/77 (20.8%)	37/264 (14.0%)	0.15	9/45 (20.0%)	44/296 (14.9%)	0.38	17/90 (18.9%)	36/251 (14.3%)	0.31
Males	11/40 (27.5%)	24/255 (9.4%)	0.003	9/27 (33.3%)	26/268 (9.7%)	0.002	12/49 (24.5%)	23/246 (9.3%)	0.003
Total	27/117 (23.1%)	61/519 (11.8%)	0.001	18/72 (25.0%)	70/564 (12.4%)	0.003	29/139 (20.9%)	59/497 (11.9%)	0.007
	**Right hip acetabular dysplasia (LCEA)**			**Left hip acetabular dysplasia (LCEA)**			**Right and/or left hip acetabular dysplasia (LCEA)**		
	**Yes**	**No**	***p*** **value**	**Yes**	**No**	***p*** **value**	**Yes**	**No**	***p*** **value**
**Right hip pain at physical examination**									
Females	5/38 (13.2%)	31/303 (10.2%)	0.58	6/28 (21.4%)	30/313 (9.6%)	0.05	7/48 (14.6%)	29/293 (9.9%)	0.34
Males	4/20 (20.0%)	24/275 (8.7%)	0.10	3/12 (25.0%)	25/283 (8.8%)	0.09	5/24 (20.8%)	23/271 (8.5%)	0.05
Total	9/58 (15.5%)	55/578 (9.5%)	0.15	9/40 (22.5%)	55/596 (9.2%)	0.007	12/72 (16.7%)	52/564 (9.2%)	0.06
**Left hip pain at physical examination**									
Females	5/38 (13.2%)	27/303 (8.9%)	0.40	3/28 (10.7%)	29/313 (9.3%)	0.74	5/48 (10.4%)	27/293 (9.2%)	0.79
Males	4/20 (20.0%)	16/275 (5.8%)	0.04	3/12 (25.0%)	17/283 (6.0%)	0.04	5/24 (20.8%)	15/271 (5.5%)	0.02
Total	9/58 (15.5%)	43/578 (7.4%)	0.04	6/40 (15.0%)	46/596 (7.7%)	0.10	10/72 (13.9%)	42/564 (7.4%)	0.06
**Hip pain at physical examination**									
Females	8/38 (21.1%)	45/303 (14.9%)	0.32	7/28 (25.0%)	46/313 (14.7%)	0.15	10/48 (20.8%)	43/293 (14.7%)	0.28
Males	6/20 (30.0%)	29/275 (10.5%)	0.02	4/12 (33.0%)	31/283 (11.0%)	0.04	7/24 (29.2%)	28/271 (10.3%)	0.01
Total	14/58 (24.1%)	74/578 (12.8%)	0.02	11/40 (27.5%)	77/596 (12.9%)	0.01	17/72 (23.6%)	71/564 (12.6%)	0.01

The data are numbers (percentages). Categorical variables were compared using the χ^2^ test or Fisher’s exact test, as appropriate

*AA* Acetabular angle, *FHEI* Femoral head extrusion index, *LCEA* Lateral centre-edge angle

^a^At least one parameter consistent with acetabular dysplasia among Tönnis angle, AA, LCEA, and FHEI

## Discussion

This study provides the first evidence that mild acetabular dysplasia in adults may be associated with hip pain upon physical examination in patients with suspected axSpA, in the same way it has been previously associated with self-reported hip pain in the general population. Hip pain was present in twice as many patients with vs. without acetabular dysplasia. Thus, the presence of acetabular dysplasia may carry a risk of overestimating hip involvement related to axSpA, in such population subject to systematic and repeated musculoskeletal evaluations.

Previous studies have established that acetabular dysplasia is common among adults and can be the sole reason for hip pain [6, 8, 20, 21]. Conflicting results have been reported [22] but are probably ascribable to differences in the definition and assessment methods of acetabular dysplasia and of hip pain. We used a standardised method for assessing hip pain and had two readers evaluate reproducible radiological signs of acetabular dysplasia. Our results support an association between acetabular dysplasia and hip pain, in the absence of clinically significant radiological hip osteoarthritis.

In our study, 22% of patients had at least one of the four radiological signs of acetabular dysplasia but only 11% had LCEA< 20°, in keeping with earlier data in various geographic areas and ethnic groups [23]. Acetabular dysplasia was more common in females, for both the right and the left hips, in agreement with other studies [16]. This was the only significant difference between the groups with vs. without acetabular dysplasia. Notably, no significant differences were shown in the parameters assessing disease activity and functional impairment in relation to axial SpA, which is hardly surprising given the global nature of these tools.

The association between acetabular dysplasia and hip pain at the patient level was confirmed at the hip level and persisted when LCEA< 20° was required to define acetabular dysplasia. The prevalence of acetabular dysplasia increased with the severity of the hip pain. The low prevalence of radiological osteoarthritis may explain the absence of association with pain or acetabular dysplasia. We found no differences across age groups but the small number of patients in each category resulted in limited statistical power, and our population was relatively young (18–50 years at enrolment).

The association between acetabular dysplasia and hip pain was significant in males but not in females. We suggest two hypotheses to explain this finding. First, conditions other than acetabular dysplasia and likely to be responsible for hip pain, such as fibromyalgia, may be more common in females [24]. However, hip pain was not significantly more common in females than in males in our study. Second, the same cut-offs were used for acetabular dysplasia in males and females. Conceivably, different cut-offs might be appropriate, and we may therefore have underestimated the prevalence of acetabular dysplasia among females [16].

Analyses at hip level showed some counter-intuitive results regarding the association between acetabular dysplasia at hip level and contralateral pain upon physical examination. For example, the results highlighted a strong association between right hip acetabular dysplasia and left hip pain among men, more marked than with the ipsilateral hip. Beyond type I error, there is no unequivocal explanation to these intriguing findings. This potentially illustrates the limits of the current definition of acetabular dysplasia, based on several parameters which are assessed binary, not accounting for global hip morphology and not accounting for subtle morphological differences that exist between right and left hip, at population level [21]. It may also illustrates the complexity of the relationship that exists between hip morphology and symptoms, that is likely to be influenced by multiples parameters, including activities and laterality. At the end, such results suggest that the presence of one or more abnormal parameters consistent with acetabular dysplasia accounts for a relevant disorder at the individual level just as much or even more than at joint level.

This study has several limitations. First, data on the characteristics of hip pain are limited, because the DESIR cohort was designed to assess axSpA. We assessed only hip pain elicited during a physical examination by a senior rheumatologist. No data were available on self-reported hip pain or on features suggesting inflammatory vs. mechanical hip pain. As a result, the possibility of an underlying coxitis in some participants cannot be formally ruled out. Regarding this last point, however, a study has shown that acetabular dysplasia can be responsible for pain at night [25]. Second, we did not have information on other common conditions potentially responsible for hip pain, such as impingement syndrome [26]. Finally, among the many parameters that have been used to diagnose acetabular dysplasia on standard antero-posterior radiographs, we selected only four. More specifically, we did not assess anterior coverage of the femoral head.

In conclusion, among males with recent-onset IBP suggesting axSpA, acetabular dysplasia was associated with hip pain upon physical examination. Thus, the common disorder of acetabular dysplasia may mistakenly suggest hip involvement by axSpA in patients with recent-onset IBP. Acetabular dysplasia should be sought routinely on radiographs in patients with suspected axSpA. Studies are needed to further characterise the association between acetabular dysplasia and hip pain and its potential effects on the diagnosis of axSpA.

## Data Availability

The study data are available upon reasonable request to the principal DESIR-cohort investigator Maxime Dougados, Paris (maxime.dougados@aphp.fr).
